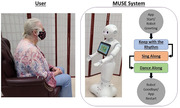# User Perceptions of a Robot‐based Music Intervention System for Dementia Care

**DOI:** 10.1002/alz.090814

**Published:** 2025-01-09

**Authors:** Tyler Morris, Sydney P. Walker, Laython V. Holder, Eric Vaughan, Darina Petrovsky, Sai Swaminathan, Xiaopeng Zhao

**Affiliations:** ^1^ University of Tennessee, Knoxville, TN USA; ^2^ Duke University, Durham, NC USA

## Abstract

**Background:**

To address the rapid increase in the number of persons with Alzheimer’s disease or related dementia (PwADRD), we seek to combine the benefits of music intervention with the adaptability of social robotics. Our system, the Music intervention Using Socially Engaging robotics (MUSE) system, seeks to provide a structured music intervention session to a group of PwADRD using the social robot Pepper. As seen in Figure 1, the Pepper robot leads the PwADRD through a 3‐step music intervention session. To assess the user perception of the MUSE system, we conducted two workshops with PwADRD.

**Method:**

We gathered groups of PwADRD with mild to moderate Alzheimer’s/dementia at assisted living facilities in the Knoxville, Tennessee area. Within these groups, the PwADRD participated in a MUSE session guided by the social robot. Afterward, they filled out a brief survey (5‐point Likert Scale questions) and participated in a semi‐structured interview.

**Result:**

From the workshops conducted, we had a total of 15 participants. Regarding Pepper robot, the average survey score for all questions was 3.58/5 with a standard deviation of 1.12. These questions covered various design aspects of the robot’s design. Furthermore, the survey showed positive results for the MUSE app. Covering the activities of the MUSE session, participants gave an average score of 3.86/5 with a standard deviation of 0.89. Additionally, in the interview, participants proclaimed their admiration for the system. However, most participants did note that it was difficult to hear the robot at times and that the tablet interface was difficult to see.

**Conclusion:**

The workshops showed that the MUSE system was positively received by the target community. As the research team observed participants engaging with the system, we found that most PwADRD enjoyed the musical activities and the robot that presented them. However, we also found ways to improve the MUSE system. Moving forward, we seek to address these issues and hopefully improve the user acceptance of the MUSE. Nonetheless, the workshops prove that the MUSE system can and will be positively received by the PwADRD community and therefore is a viable way to provide a meaningful intervention to PwADRD.